# Impact of postoperative depression and immune-inflammatory biomarkers on the prognosis of patients with esophageal cancer receiving minimally invasive esophagectomy: a retrospective cohort study based on a Chinese population

**DOI:** 10.3389/fimmu.2025.1610267

**Published:** 2025-06-06

**Authors:** Pei-xin Tan, Lin-xin Wu, Shuang Ma, Shi-jing Wei, Tai-hang Wang, Bing-chen Wang, Bing-bing Fu, Jia-shuo Yang, Qing Zhao, Li Sun, Yi Liu, Tao Yan

**Affiliations:** ^1^ Department of Anesthesiology, National Cancer Center/National Clinical Research Center for Cancer/Cancer Hospital, Chinese Academy of Medical Sciences and Peking Union Medical College, Beijing, China; ^2^ School of Information Science and Engineering, Shenyang Ligong University, Shenyang, China; ^3^ Department of Hepatobiliary Surgery, National Cancer Center/National Clinical Research Center for Cancer/Cancer Hospital, Chinese Academy of Medical Sciences and Peking Union Medical College, Beijing, China; ^4^ Department of Diagnostic Radiology, National Cancer Center/National Clinical Research Center for Cancer/Cancer Hospital, Chinese Academy of Medical Sciences and Peking Union Medical College, Beijing, China; ^5^ Department of Radiology, Shanxi Province Cancer Hospital/Shanxi Hospital Affiliated to Cancer Hospital, Chinese Academy of Medical Sciences/Cancer Hospital Affiliated to Shanxi Medical University, Taiyuan, China; ^6^ Department of Anesthesiology, Shenzhen Samii Medical Centre, The Fourth People’s Hospital of Shenzhen, Shenzhen, China; ^7^ Department of Anesthesiology, Shanxi Province Cancer Hospital/Shanxi Hospital Affiliated to Cancer Hospital, Chinese Academy of Medical Sciences/Cancer Hospital Affiliated to Shanxi Medical University, Taiyuan, China

**Keywords:** esophageal cancer, prognosis, depression, immune-inflammatory biomarkers, predictive model

## Abstract

**Background:**

Patients with esophageal cancer (EC) frequently experience depression following neoadjuvant therapy and surgery, a condition that may trigger systemic inflammation, suppress antitumor immunity, and alter immune-inflammatory pathways in the tumor microenvironment (TME), potentially contributing to residual tumor progression and theoretically worsening patient prognosis. This study aimed to investigate the interrelationship between depression and prognosis in patients with EC, with a focus on immune-inflammatory biomarkers.

**Methods:**

This single-center retrospective trial was conducted at the National Cancer Center/Cancer Hospital of the Chinese Academy of Medical Sciences. A total of 319 patients who underwent minimally invasive esophagectomy between November 2023 and December 2024 were enrolled. Least absolute shrinkage and selection operator (LASSO) regression in combination with multivariate Cox and logistic regression were employed to identify the main impact indicators of relapse-free survival (RFS) and depression. The developed predictive model was evaluated using calibration plots, receiver operating characteristic (ROC) curves, and decision curve analysis (DCA). Internal validation was carried out using a 7:3 data split.

**Results:**

LASSO and Cox regression identified clinical stage (hazard ratio [HR]=2.472, P=0.003), the preoperative systemic inflammatory index (SII, HR=1.001, P<0.001), and depression severity (HR=2.398, P=0.004) as independent predictors of RFS. Based on these variables, a predictive model for RFS was constructed utilizing multivariate logistic regression and visualized as a nomogram. The model demonstrated good discriminative ability, with the areas under the ROC curves (AUCs) of 0.826 (6 months) and 0.773 (12 months) in the training set and 0.817 (6 months) and 0.789 (12 months) in the validation set. The incidence of postoperative depression in the study cohort was 28.2%, with chronic postsurgical pain identified as the sole independent risk factor for depression.

**Conclusion:**

This study revealed that preoperative immune-inflammatory biomarkers and postoperative depression significantly affect patient prognosis after minimally invasive esophagectomy. Our work has also provided new insight into the individualized and comprehensive management of patients with EC, underscoring the necessity for comprehensive psychosocial interventions alongside conventional anticancer therapies to optimize clinical endpoints.

## 1 Introduction

According to the 2024 GLOBOCAN report, esophageal cancer (EC) ranks 7th in terms of global cancer-related mortality and remains one of the most prevalent malignant tumors, with a 5-year survival rate ranging from 10% to 30% after diagnosis (1, 2). In 2022, there were approximately 511,000 new cases of EC worldwide, resulting in an estimated 445,000 deaths. Notably, China accounts for more than 50% of the global burden, with 346,000 new cases and 323,000 deaths (1, 3). With advancements in endoscopic surgery techniques, minimally invasive esophagectomy (MIE) has emerged as the primary treatment for EC and substantially improves patient survival. Nevertheless, given the highly traumatic nature of the procedure, prolonged postoperative recovery period, and increased rate of postoperative complications, patients with EC usually suffer from poor postoperative quality of life and long-term health outcomes (4–10).

Depression is a major complication among postoperative patients with cancer (5). The postoperative depression rate among patients with breast cancer might exceed 30% (11), and a recent meta-analysis revealed that depression might predict breast cancer mortality (12). In the case of lung cancer, the morbidity rates of patients with newly developed depression after thoracoscopic surgery and open thoracotomy are 12.4% and 16.1%, respectively (13). Patients with EC also experience a relatively high incidence of depression, with rates of approximately 20% preoperatively and 27% at 6 months and 32% at 12 months postoperatively (14). These statistics underscore the considerable psychological burden faced by patients with cancer throughout the continuum of care.

In addition to the well-documented psychological burden of depression, emerging evidence suggests that its pathophysiology may be intertwined with systemic inflammatory processes (15). Systemic inflammation can modulate the comorbidity of depression and impact tumor progression through neuroimmune pathways (16) and the tumor microenvironment (TME) (17). Conversely, inflammatory mediators such as tumor necrosis factor-α (TNF-α), interleukin-1β (IL-1β) and IL-6 can be released in response to psychological stress and surgical injury and can cross the blood-brain barrier and induce the upregulation of corticotropin-releasing factor (CRF) (18). Concurrently, these inflammatory signals cause dysfunction of the reward circuit (19), which further exacerbates emotional disorders, thereby creating a vicious cycle.

The relationship between postoperative depression and cancer prognosis has attracted increasing attention, with recent investigations emphasizing the role of underlying inflammatory mechanisms (20) and advances in the biopsychosocial model (21). MIE involves significant surgical trauma, complex multimodal therapies, and extended recovery periods, all of which impose considerable biological and psychological stress on patients. EC exhibits a lower prevalence outside Asia, and the evidence linking depression to survival outcomes remains limited (5, 22). In light of this gap, we conducted a retrospective analysis to evaluate the association between depression and relapse-free survival (RFS) and to explore the potential role of perioperative immune-inflammatory biomarkers in mediating this association.

## 2 Materials and methods

### 2.1 Patient selection

In this retrospective cohort study, we enrolled patients with EC at the National Cancer Center/Cancer Hospital of the Chinese Academy of Medical Sciences from November 2023 to December 2024. Participants were included based on the following criteria: (1) pathological diagnosis of esophageal adenocarcinoma (EAC), esophageal squamous cell carcinoma (ESCC), or esophageal adenosquamous carcinoma; (2) underwent MIE; (3) at least 3 months of postoperative follow-up with complete follow-up data available; and (4) at least 18 years of age and capable of understanding the study objectives to comply with the follow-up procedures.

The exclusion criteria were as follows: (1) the presence of distant metastasis (defined as M1 stage according to the 8th edition of the AJCC TNM classification) or concomitant malignant tumors before esophagectomy; (2) a history of chronic pain (defined as pain persisting for more than 3 months, including chronic low back pain, arthritis-related pain, or neuropathic pain) or a preoperative diagnosis of a mental disorder (including major depressive disorder, generalized anxiety disorder, bipolar disorder, or schizophrenia); (3) the presence of severe infection, hematologic disease, or autoimmune disease; and (4) incomplete clinical data or a follow-up duration of less than 3 months.

Ethics approval was received from the Institutional Ethics Committee of Cancer Hospital (Approval Number: 25/110-5056). All data were extracted from the database of the clinical electronic medical records system. Medical ethical principles were strictly observed during the research process to ensure patient privacy and data confidentiality, and all data were used only for analysis and reports in this study.

### 2.2 Data acquisition

Data on population characteristics and clinical aspects, including age, sex, height, weight, body mass index (BMI), history of neoadjuvant therapy, tumor type, clinical stage, tumor differentiation, postoperative adjuvant therapy, and other relevant data, were retrieved from the clinical electronic medical records system. Postoperative pathology results were assessed through pathological reports (ypTNM by the American Joint Committee on Cancer, 8^th^ edition) (23). Hematologic parameters, including neutrophils (NEU) count, lymphocytes (LYM) count, monocytes (MONO) count, albumin (ALB) level, C-reactive protein (CRP) level, and platelets (PLT) count, were collected preoperatively and one week postoperatively. Immune-inflammatory biomarkers were processed using the following calculations: (1) systemic immune-inflammation index (SII) =PLT×NEU/LYM; (2) systemic inflammation response index (SIRI) =NEU×MONO/LYM; (3) neutrophil-to-lymphocyte ratio (NLR) =NEU/LYM; (4) inflammatory burden index (IBI) =CRP×NEU/LYM; (5) pan-immune-inflammation value (PIV) =NEU×MONO×PLT/LYM; (6) CRP-to-albumin ratio (CAR) =CRP/ALB; (7) CRP-to-lymphocyte ratio (CLR) =CRP/LYM; (8) lymphocyte-to-monocyte ratio (LMR) =LYM/MONO; and (9) CRP-albumin-lymphocyte (CALLY) index =ALB×LYM/(CRP×10).

### 2.3 Follow-up

All patients received postoperative follow-up through outpatient visits or telephone conversations, with assessments conducted every three months. The follow-up period lasted from the date of surgery to March 30, 2025, or until all-cause mortality. We defined the RFS as the interval from surgery to confirmed disease recurrence. Recurrence or metastasis was diagnosed based on pathologic examination or imaging modalities with diagnostic value, including computed tomography (CT), magnetic resonance imaging (MRI), bone scintigraphy and others.

A psychiatrist with specialized expertise evaluated the patient’s postoperative depression using the Patient Health Questionnaire (PHQ-9) (**Supplementary Table 1**). Follow-up visits were conducted between 9:00 AM and 10:00 AM at 3 months postoperatively to ensure the homogeneity of the follow-up data. The overall score of nine items (each rated on a scale of 0–3) was calculated, with higher scores indicating a greater level of depression (24, 25). The total PHQ-9 total score ranges from 0 to 27 (scores of 0–4 are classified as nondepression; scores of 5–9 are classified as mild depression; and scores of ≥10 are classified as moderate-to-severe depression) (26, 27).

An experienced anesthesiologist assessed postoperative pain using the numeric rating scale (NRS) (28) at 48 h and 3 months after surgery. Pain that emerged or intensified following surgical procedures, persisted for more than 3 months, and was confined to the surgical area was regarded as chronic postsurgical pain (CPSP), as defined by the International Association of the Study of Pain (29).

### 2.4 Statistical analysis

The datasets used in the study were analyzed using R software (version 4.2.1; R Foundation for Statistical Computing) and IBM SPSS Statistics 26.0 software. The tidyverse package (version 1.3.1) in R was deployed for multiple imputations to handle missing data. The caret package (versions 6.0-90) was used to randomly assign patients to a 7:3 split, with 70% in the training set and 30% in the validation set for internal validation. Data with a normal distribution are presented as the mean ± standard deviation. To compare two groups, Student’s t-test was employed, and three or more groups were compared using one-way ANOVA. Data that did not adhere to the normal distribution are presented as the median (quartile range). Comparisons between two groups were performed with the Wilcoxon rank sum test, and comparisons among three or more groups were performed with the Kruskal-Wallis test. Count data are expressed as frequencies (%), and differences were assessed via the χ^2^ test or Fisher’s exact test.

We employed the R language ggplot2 package to draw survival curves (version 3.4.0) and the R language survival package to perform the log-rank test (version 3.5-7). The variables were subsequently screened using the least absolute shrinkage and selection operator (LASSO) and cross-validated with 10-fold partitioning. LASSO regression was selected for variable screening because it is particularly effective in handling high-dimensional data and multicollinearity while preventing model overfitting, which aligns well with the characteristics of our dataset. We determined the optimal regularization parameter λ by performing 10-fold cross-validation and selecting the largest λ value within one standard error of the minimum cross-validation error (λ1se). The glmnet package (version 4.1-4) was used to conduct the LASSO regression. Multivariate Cox regression analysis was used to evaluate the independent effects of multiple variables on survival and control for the effects of other confounding factors. The hazard ratio (HR) was calculated for each predictive factor, along with the corresponding 95% confidence interval (CI). To construct a predictive model, multivariate logistic regression was applied to the variables that had been screened previously. The model was then visualized by constructing a nomogram for RFS. To determine the effectiveness of the model, the receiver operating characteristic (ROC) curve was drawn, and the areas under the ROC curves (AUCs) were computed. In addition, the accuracy of its prediction and clinical utility were further evaluated by employing calibration curves and decision curve analysis (DCA). The analysis and figures were obtained using the proc (v1.18.0), rms (v6.3-0), and rmda (v1.6.0) packages. A P value less than 0.05 was regarded as statistically significant unless otherwise noted.

## 3 Results

**Figure 1:**
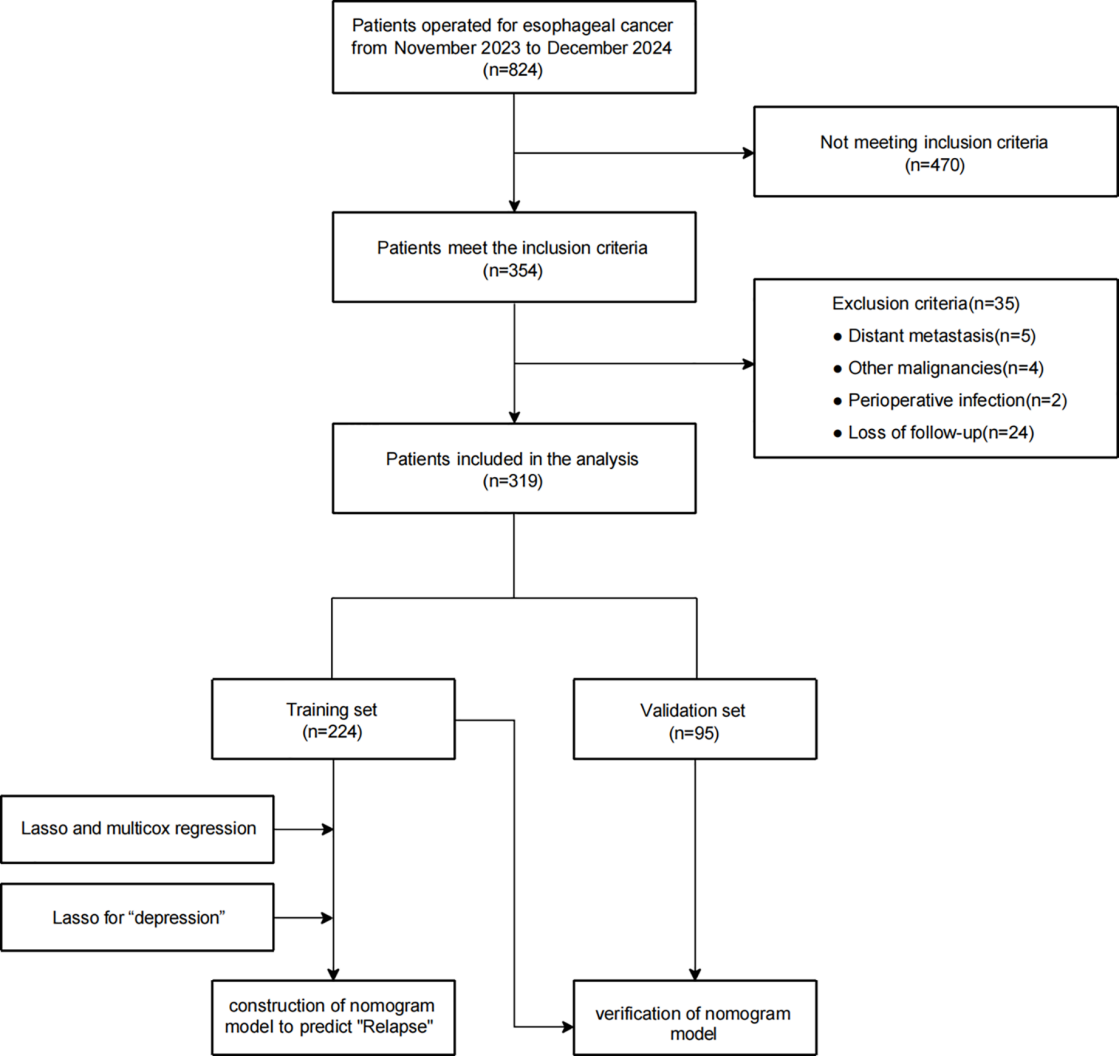
Inclusion and exclusion flow chart.

Based on the inclusion criteria, 354 patients were enrolled from the initial 824 patients, 35 of whom were excluded for meeting exclusion criteria, including other surgical procedures (due to distant metastasis or other malignancies), surgical contradictions (due to perioperative infection, blood disease, or autoimmune disease), or loss to follow-up. Ultimately, the analysis included 319 patients. **Figure 1** illustrates the criteria for including or excluding patients who underwent surgery for EC between November 2023 and December 2024. For further analysis, these patients were split into two categories: 224 for training and 95 for validation.

### 3.1 Baseline demographics

To confirm the consistency of the significant baseline characteristics, comparisons were made between the baseline variables of the training and validation sets. There were 95 patients in the validation set and 224 in the training set, with an allocation ratio of 3:7. According to the statistical test results, the baseline features of the patients in the validation and training sets did not differ significantly in any other way except for the status of proficient mismatch repair (pMMR) (P=0.004, **Table 1**).

**Table 1:** Statistical test results of training set and validation set.

Characteristic	Whole population (n=319)	Training cohort (n=224)	Validation cohort (n=95)	P value
Sex ^a^				0.431
Female	43 (13.5)	28 (12.5)	15 (15.8)	
Male	276 (86.5)	196 (87.5)	80 (84.2)	
Age ^b^	63.2 ± 7.6	63.2 ± 7.8	63.2 ± 7.1	0.983
BMI ^b^	23.4 ± 3.1	23.3 ± 3.2	23.7 ± 2.9	0.243
Neoadjuvant chemotherapy ^a^				0.870
No	72 (22.6)	50 (22.3)	22 (23.2)	
Yes	247 (77.4)	174 (77.7)	73 (76.8)	
Neoadjuvant immunotherapy ^a^				0.629
No	107 (33.5)	77 (34.4)	30 (31.6)	
Yes	212 (66.5)	147 (65.6)	65 (68.4)	
Neoadjuvant radiotherapy ^a^				0.773
No	278 (87.1)	196 (87.5)	82 (86.3)	
Yes	41 (12.9)	28 (12.5)	13 (13.7)	
Pathological response ^a^				0.760
non-neoadjuvant therapy	224 (70.2)	160 (71.4)	64 (67.4)	
pCR	47 (14.7)	32 (14.3)	15 (15.8)	
Non-pCR	48 (15)	32 (14.3)	16 (16.8)	
Chemotherapy cycles ^a^				0.962
0 cycles	74 (23.2)	52 (23.2)	22 (23.2)	
1–3 cycles	194 (60.8)	137 (61.2)	57 (60)	
≥ 4 cycles	51 (16)	35 (15.6)	16 (16.8)	
Nausea ^a^				0.177
No	296 (92.8)	205 (91.5)	91 (95.8)	
Yes	23 (7.2)	19 (8.5)	4 (4.2)	
Dizzy ^a^				0.440
No	282 (88.4)	196 (87.5)	86 (90.5)	
Yes	37 (11.6)	28 (12.5)	9 (9.5)	
NRS (at rest) ^a^				0.135
Mild pain	299 (93.7)	207 (92.4)	92 (96.8)	
Moderate-high pain	20 (6.3)	17 (7.6)	3 (3.2)	
NRS (during activity) ^a^				0.881
Mild pain	240 (75.2)	168 (75)	72 (75.8)	
Moderate-high pain	79 (24.8)	56 (25)	23 (24.2)	
PDL1 positivity ^a^				0.673
(-)	247 (77.4)	172 (76.8)	75 (78.9)	
(+)	72 (22.6)	52 (23.2)	20 (21.1)	
pMMR ^a^				0.004
No	289 (90.6)	196 (87.5)	93 (97.9)	
Yes	30 (9.4)	28 (12.5)	2 (2.1)	
Pathology ^a^				0.621
Non-ESCC	36 (11.3)	24 (10.7)	12 (12.6)	
ESCC	283 (88.7)	200 (89.3)	83 (87.4)	
Differentiation ^a^				0.699
poorly differentiated	131 (41.1)	95 (42.4)	36 (37.9)	
moderately differentiated	153 (48)	103 (46)	50 (52.6)	
well-differentiated	14 (4.4)	11 (4.9)	3 (3.2)	
Intraepithelial Neoplasia	21 (6.6)	15 (6.7)	6 (6.3)	
Clinical stage ^a^				0.323
I+II	142 (44.5)	99 (44.2)	43 (45.3)	
III	157 (49.2)	108 (48.2)	49 (51.6)	
IV	20 (6.3)	17 (7.6)	3 (3.2)	
Preoperative SII ^c^	409.1 (279.5, 628.3)	406.8 (283.6, 615.3)	419.7 (266.1, 648.6)	0.564
Preoperative SIRI ^c^	0.9 (0.6, 1.3)	0.9 (0.6, 1.3)	0.9 (0.6, 1.2)	0.864
Preoperative NLR ^c^	2.2 (1.6, 3.2)	2.2 (1.6, 3.3)	2.3 (1.5, 3)	0.941
Preoperative PIV ^c^	165.3 (100.7, 268.2)	165.1 (100.1, 264.8)	170.6 (102.9, 273.5)	0.902
Preoperative IBI ^c^	3.1 (1.3, 8.6)	3.1 (1.2, 9.8)	3 (1.4, 7.7)	0.548
Preoperative CAR ^c^	0 (0, 0.1)	0 (0, 0.1)	0 (0, 0.1)	0.431
Preoperative CLR ^c^	1 (0.4, 3.6)	1.1 (0.3, 4.4)	1 (0.4, 1.9)	0.647
Preoperative LMR ^c^	3.9 (2.9, 5.2)	3.9 (2.9, 5.3)	3.9 (2.8, 5.1)	0.782
Preoperative CALLY ^c^	4.3 (1.8, 10.7)	4.1 (1.6, 10.3)	4.4 (2.4, 11.7)	0.259
Postoperative SII ^c^	914.1 (663.9, 1364.6)	939.7 (686.6, 1360.5)	900.5 (617.7, 1347.2)	0.582
Postoperative SIRI ^c^	2 (1.4, 3.3)	2.1 (1.4, 3.4)	1.9 (1.3, 3.2)	0.292
Postoperative NLR ^c^	4.6 (3.4, 6.8)	4.7 (3.4, 6.8)	4.3 (3.2, 6.6)	0.430
Postoperative PIV ^c^	431.2 (263.8, 701.5)	444 (269.5, 724.5)	398 (259.6, 591.1)	0.326
Postoperative IBI ^c^	27 (12.3, 53.7)	29.3 (12.9, 53.9)	21.2 (11.4, 44)	0.090
Postoperative CAR ^c^	0.1 (0.1, 0.3)	0.2 (0.1, 0.3)	0.1 (0.1, 0.3)	0.136
Postoperative CLR ^c^	5.3 (2.8, 10.5)	5.5 (3.1, 11.1)	4.6 (2.5, 9.2)	0.196
Postoperative LMR ^c^	2.3 (1.7, 3.1)	2.3 (1.7, 3)	2.4 (1.7, 3.3)	0.515
Postoperative CALLY ^c^	0.1 (0, 0.1)	0.1 (0, 0.1)	0.1 (0, 0.2)	0.237
Distance from incisors ^a^				0.906
Upper	136 (42.6)	97 (43.3)	39 (41.1)	
Middle	168 (52.7)	117 (52.2)	51 (53.7)	
Lower	15 (4.7)	10 (4.5)	5 (5.3)	
Postoperative chemotherapy ^a^				0.316
No	212 (66.5)	145 (64.7)	67 (70.5)	
Yes	107 (33.5)	79 (35.3)	28 (29.5)	
Postoperative immunotherapy ^a^				0.765
No	228 (71.5)	159 (71)	69 (72.6)	
Yes	91 (28.5)	65 (29)	26 (27.4)	
Postoperative radiotherapy ^a^				0.052
No	286 (89.7)	196 (87.5)	90 (94.7)	
Yes	33 (10.3)	28 (12.5)	5 (5.3)	
Depression severity ^a^				0.413
Nondepressed	229 (71.8)	157 (70.1)	72 (75.8)	
Mild depressed	79 (24.8)	60 (26.8)	19 (20)	
Moderate-to-severe depressed	11 (3.4)	7 (3.1)	4 (4.2)	
CPSP ^a^				0.767
No	222 (69.6)	157 (70.1)	65 (68.4)	
Yes	97 (30.4)	67 (29.9)	30 (31.6)	

a Data are n (%) and are compared by χ^2^ test.

b Data are mean ± standard deviation and are compared by Student’s t test.

c Data are median (interquartile range) and are compared by Mann-Whitney U test.

Abbreviations: BMI, body mass index; pCR, pathological complete response; pMMR, proficient mismatch repair; ESCC, esophageal squamous cell carcinoma; SII, systemic immune-inflammation index; SIRI, systemic inflammation response index; NLR, neutrophil-to-lymphocyte ratio; PIV, pan-immune-inflammation value; IBI, inflammatory burden index; CAR, C-reactive protein to albumin ratio; CLR, C-reactive protein to lymphocyte ratio; LMR, lymphocyte to monocyte ratio; CALLY, CRP-albumin-lymphocyte index.

### 3.2 Identification of independent prognostic factors

**Figure 2:**
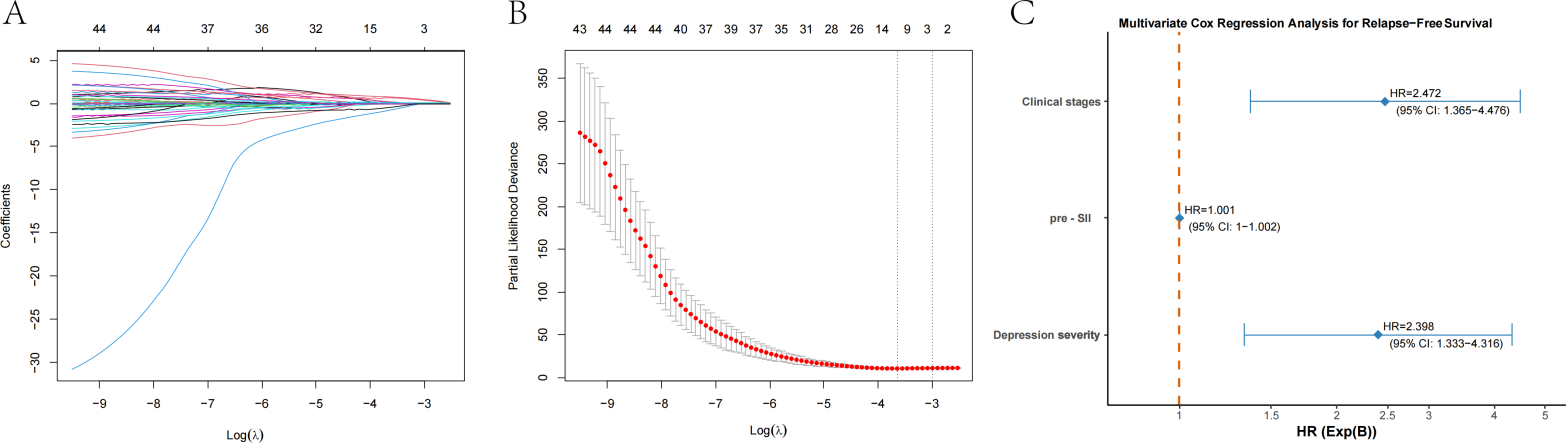
LASSO regression and multivariate Cox regression for prognostic factors in patients with esophageal cancer. **(A)** LASSO regression plot showing the relationship between the logarithm of the penalty parameter [Log (λ)] and the coefficients of selected prognostic variables. **(B)** Partial likelihood deviance plot from LASSO regression, illustrating the fit of the model as the Log (λ) changes. **(C)** Multivariate Cox regression analysis for relapse-free survival (RFS), showing the hazard ratios (HR) for clinical stage, the preoperative systemic inflammatory index (pre-SII), and depression severity. The HR values with 95% confidence intervals (CI) are provided for each factor.

We used the maximum selection rank statistics method to perform LASSO regression analysis to screen for key variables associated with RFS in patients with EC (results in **Figures 2A, B**; λ1se=3). LASSO regression identified clinical stage, preoperative SII, and depression severity as key factors affecting RFS.

These elements were subsequently incorporated into the multivariate Cox regression analysis. The clinical stage, preoperative SII, and depression severity independently predicted RFS in patients with EC. The RFS was significantly associated with clinical stage (P=0.003), and the mortality risk increased approximately 2.47-fold for each additional clinical stage (HR=2.472, 95% CI: 1.365-4.476). Among the various immune-inflammatory biomarkers considered, the SII was selected as the key predictor for RFS. The P value of the preoperative SII was less than 0.001, and for each additional unit of preoperative SII, the risk of relapse slightly increased (HR=1.001, 95% CI: 1–1.002). Depression severity was also significantly associated with RFS (P=0.004). The relapse risk in patients with severe depression was 2.4 times greater than that in patients with mild depression (HR=2.398, 95% CI: 1.333–4.316). The results were shown in **Figure 2C**.

**Figure 3:**
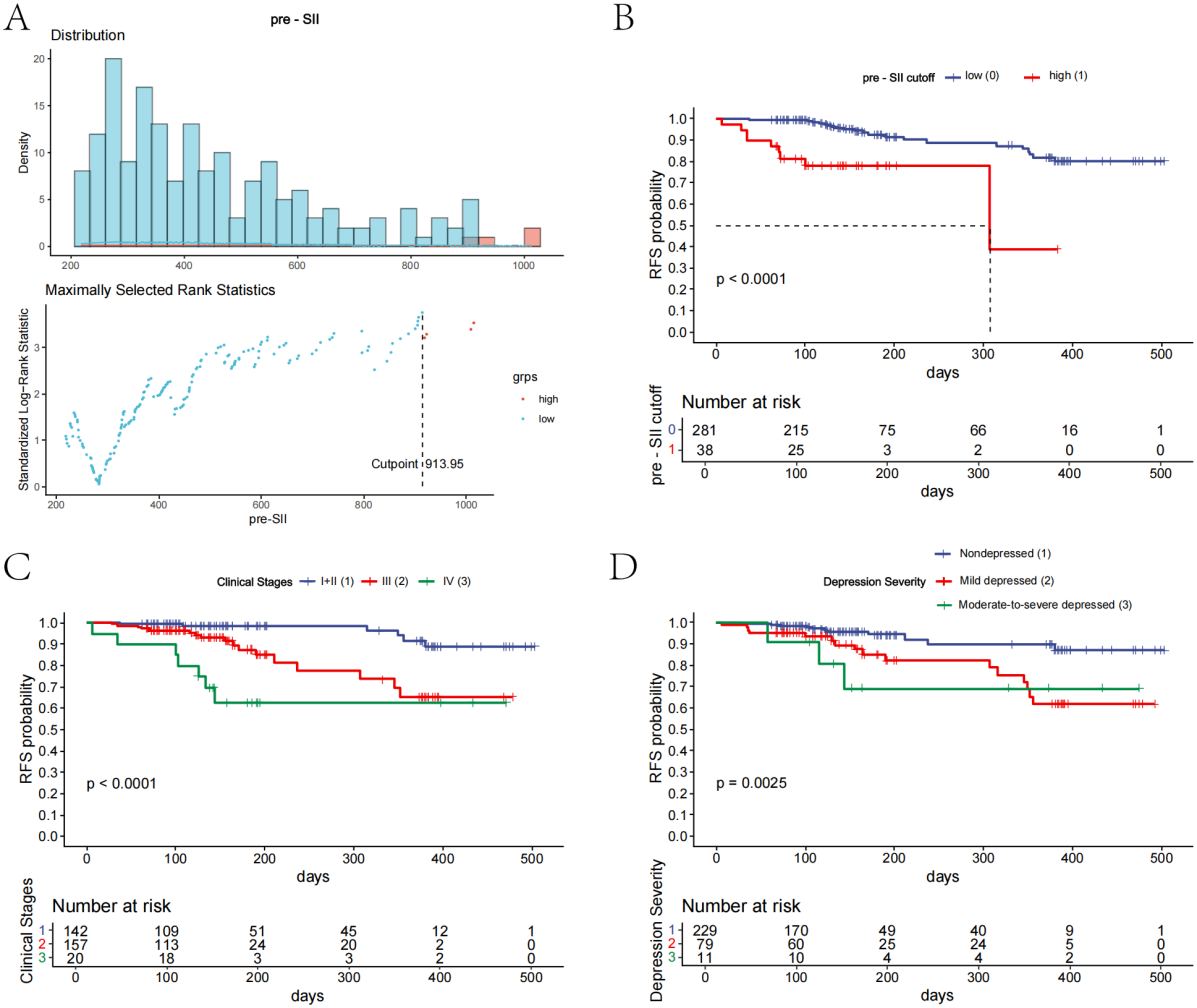
Kaplan-Meier curves for prognostic factors in patients with esophageal cancer. **(A)** The distribution of the preoperative systemic inflammatory index (pre-SII) and the optimal cutoff value of 913.95 were determined using maximally selected rank statistics. **(B)** Kaplan-Meier curve illustrating the relapse-free survival (RFS) for patients with a preoperative SII above or below the established cutoff value. **(C)** Kaplan-Meier curve showing the RFS for patients stratified by clinical stage. **(D)** Kaplan-Meier curve showing the RFS for patients categorized by depression severity.

We determined the optimal cutoff point of the preoperative systemic inflammatory index (pre-SII) to be 913.95 based on the maximally selected rank statistics. Patients were classified into a low pre-SII group or a high pre-SII group in accordance with this threshold (**Figure 3A**). A further log-rank test revealed that the SII was a significant influencing factor for RFS, and compared with that of the low pre-SII group, the RFS of the high pre-SII group was substantially lower (P<0.0001; **Figure 3B**). With increasing clinical stage, the RFS of patients was notably reduced, and the difference was highly statistically significant (P<0.0001; **Figure 3C**). Patients with varying severities of depression showed that those with severe depression had the poorest RFS, with statistically significant differences (P=0.0025; **Figure 3D**). Other factors were not significantly associated with patient RFS.

### 3.3 Design and confirmation of the predictive model

**Figure 4:**
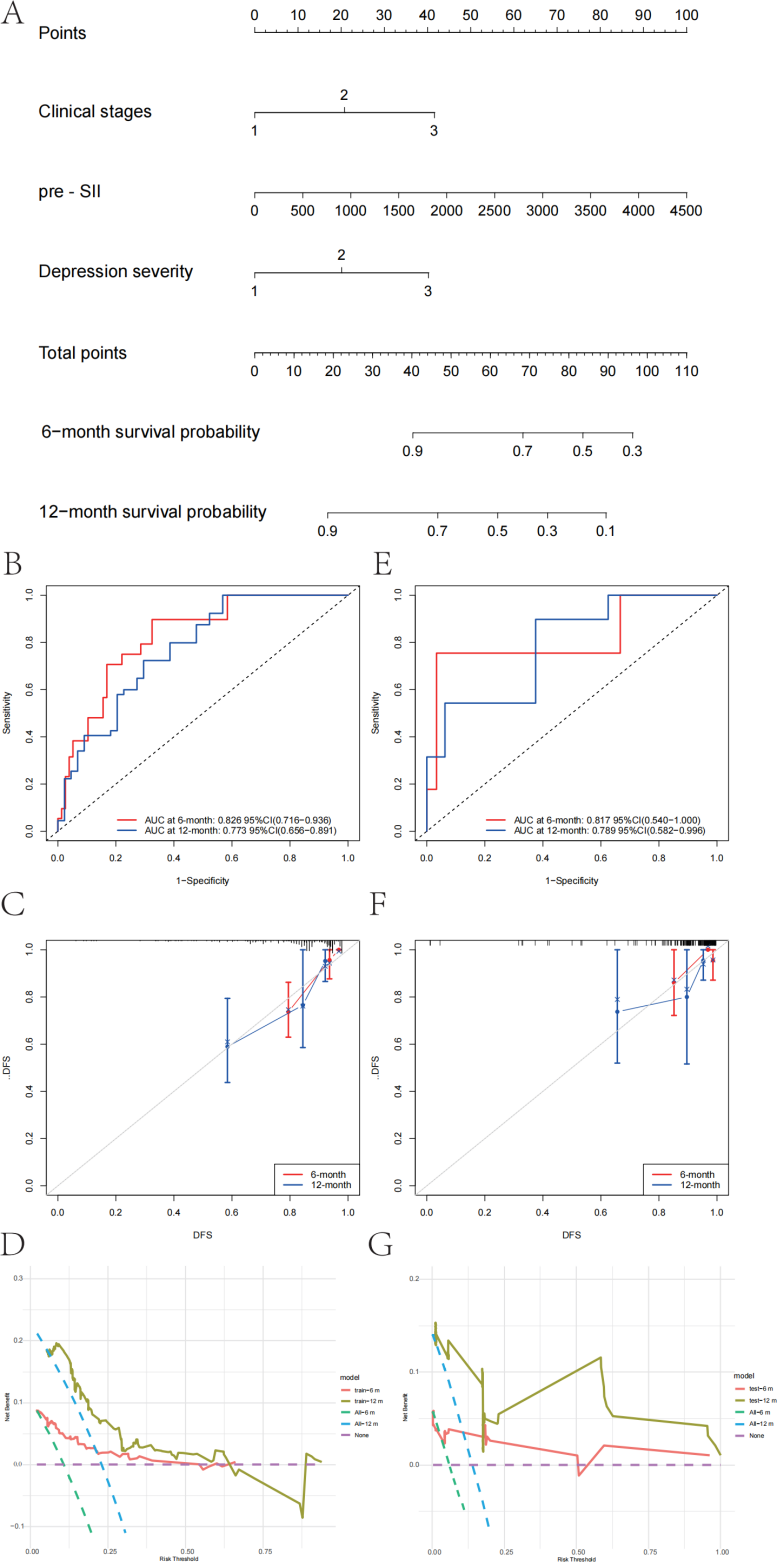
Nomogram and performance evaluation for prognosis prediction in patients with esophageal cancer. **(A)** Nomogram for predicting the 6-month and 12-month survival probabilities of patients with esophageal cancer. The total points are calculated based on the clinical stage, preoperative systemic inflammatory index (pre-SII), and depression severity. **(B)** Receiver operating characteristic (ROC) curve for the 6-month and 12-month survival predictive models in the training set. **(C)** Calibration curve for the 6-month and 12-month survival predictive models in the training set, showing the agreement between the predicted and observed survival probabilities. **(D)** Decision curve analysis (DCA) for the 6-month and 12-month survival models in the training set, evaluating the net clinical benefit of using the model at different threshold probabilities. **(E)** ROC curve for the 6-month and 12-month survival predictive models in the validation set. **(F)** Calibration curve for the 6-month and 12-month survival predictive models in the validation set, demonstrating model calibration. **(G)** DCA for the 6-month and 12-month survival models in the validation set was performed to assess the net clinical benefit of the model for decision-making.

Using the independent predictors from the multivariate Cox regression, a logistic regression model was formulated to estimate RFS risk. The nomogram in **Figure 4A** illustrates the predictive model for estimating patient survival probability at 6 months and 12 months following surgery. The performance of the model was measured by the ROC curve, with the training set’s 6-month and 12-month prediction AUCs being 0.826 (95% CI: 0.716–0.936) and 0.773 (95% CI: 0.656–0.891), respectively (**Figure 4B**). In the validation set, the AUCs for the 6-month and 12-month predictions were 0.817 (95% CI: 0.540–1.000) and 0.789 (95% CI: 0.582–0.996), respectively (**Figure 4E**). The calibration plots of the model for 6-month prediction in the training set are shown in **Figure 4C**, and the calibration plots of the model for 12-month prediction in the validation set are shown in **Figure 4F**, indicating that the nomogram was closely aligned with the observed postoperative recurrence outcomes. Further evaluation of the nomogram graph was conducted using decision curve analysis (DCA), as shown in **Figures 4D,G**. The model curves were higher than the baselines within a certain range in the training set (**Figure 4D**), especially within the threshold probability interval of 10% to 60%. Although the overall net benefit value was slightly lower in the validation set (**Figure 4G**), the model curves were still higher than the baselines of “Treat All” and “Treat None” between the 5% and 50% intervals, indicating that the model has good generalizability for external data and has certain clinical decision-making value. Our recently created nomogram can successfully differentiate between patients at high and low risk and has good discrimination capacity in both the training and validation categories, suggesting that the clinical stage, preoperative SII, and depression severity are key risk factors for EC recurrence.

### 3.4 Identification of independent influencing factors of depression

**Figure 5:**
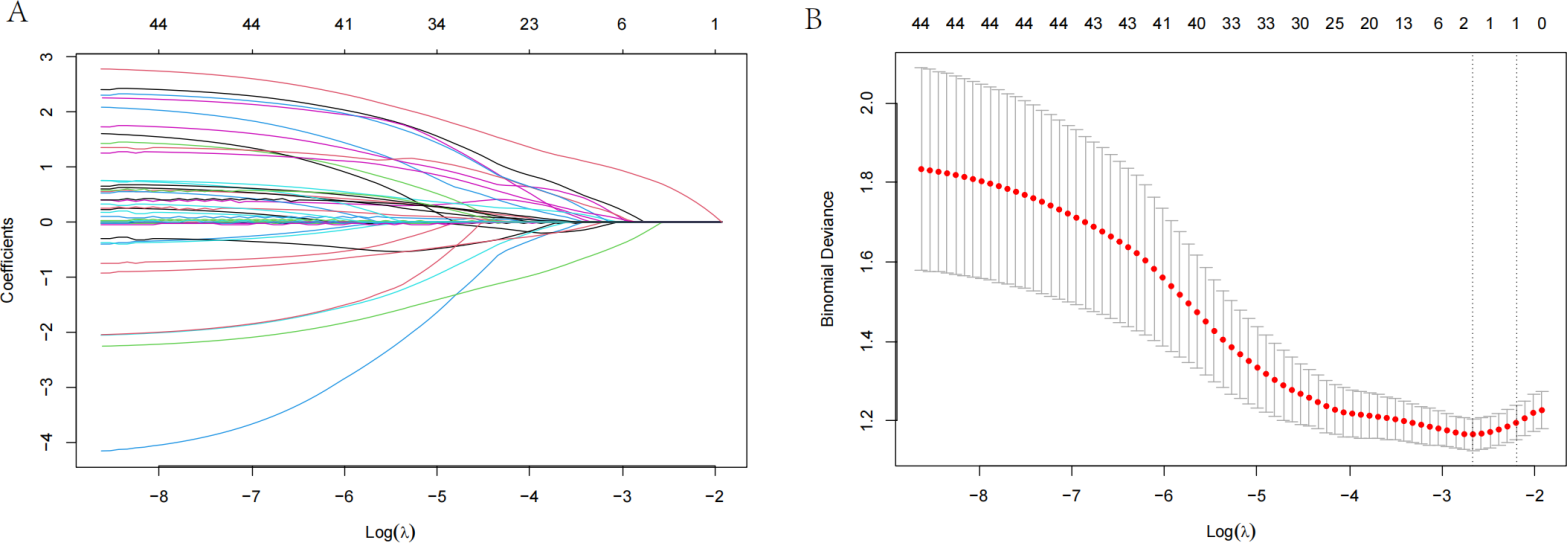
LASSO regression analysis for postoperative depression in patients with esophageal cancer. **(A)** LASSO regression plot showing the relationship between the logarithm of the penalty parameter [Log (λ)] and the coefficients of the selected variables for depression. **(B)** Partial likelihood deviance plot from LASSO regression, illustrating the fit of the model as the Log (λ) changes.

In the present study, the postoperative survival outcomes of patients were systematically evaluated, and the importance of variables such as clinical stage, inflammatory indicators, and psychological state in prognosis prediction was revealed by constructing a multivariate nomogram model. On this basis, this study focused on the prediction and analysis of postoperative depression. Among the 319 analyzed patients, 90 experienced postoperative depression (28.2%), with 79 (24.8%) classified as mild and 11 (3.4%) as moderate-to-severe based on follow-up assessments (**Table 1**). To identify the main factors influencing postoperative depression in patients with EC, we used the maximum selection rank statistics method to perform LASSO regression analysis. According to the outcomes, CPSP was the only independent risk factor for depression that was identified (results in **Figures 5A, B**; λ1se=1), precluding the possibility of a predictive model.

## 4 Discussion

This study retrospectively analyzed the correlation between depression and RFS in patients with EC and further explored the predictive value of several popular immune-inflammatory biomarkers. The results demonstrated that clinical stage, preoperative SII, and depression severity were independent prognostic factors for RFS. The predictive model, constructed based on these factors, demonstrated strong discrimination and calibration for predicting 6-month and 12-month recurrence across the training and validation cohorts, with DCA confirming its clinical utility. These findings underscore the critical prognostic value of inflammatory biomarkers alongside depression.

Consistent with earlier studies (30–33), we found that the preoperative SII was an independent factor for RFS, further confirming that inflammatory markers can be used as effective predictors of prognosis in patients with EC. A large prospective cohort study revealed that the SII was more strongly associated with cancer risk than other inflammatory markers, such as the NLR and the LMR (34). Recent studies have demonstrated that the SII, calculated as the platelet count × neutrophil count/lymphocyte count, reflects systemic immune-inflammatory conditions and is linked to both the morbidity and overall mortality of patients with cancer (35, 36). Neutrophils can promote metastasis from the primary tumor site by promoting the escape of cancer cells into the vasculature and escorting circulating tumor cells to enable cell cycle progression (37, 38). Similarly, platelets actively participate in every stage of tumorigenesis, including tumor growth, tumor cell extravasation, and metastasis (39). Conversely, both T lymphocytes and B lymphocytes are critical in the antitumor immune response (40, 41). Thus, a higher SII suggests a dominance of tumor-promoting inflammation over immune surveillance, which is correlated with a worse prognosis. Similar to a previous report, which identified a preoperative SII ≤916.6 as a favorable prognostic indicator in patients with advanced ESCC (24), an SII ≤913.95 was associated with significantly prolonged RFS in patients with EC. In addition to the SII, several other immune-inflammatory biomarkers have also been used to predict the prognosis of different types of tumors in previous studies. The CALLY index has been reported to be a positive indicator of long-term survival in patients with EC (42). Additionally, a higher CLR has also been reported to be related to negative outcomes in patients with colorectal and pancreatic cancers (43). Moreover, a multicenter prospective study revealed that the IBI is the most effective systemic inflammatory marker for predicting the outcome of non-small cell lung cancer, and patients with higher IBI levels had a notably poorer prognosis than individuals with lower IBI levels did (44). However, these factors did not correlate with prognosis in patients with EC in this study. This discrepancy may be attributed to the heterogeneity of cancer-related inflammatory pathways.

MIE involves significant surgical trauma, complex multimodal therapies, and a long recovery period, and is often associated with chronic pain, all of which put physical and psychological stress on the patient and predispose them to depression. From a clinical perspective, the prevalence of depression reportedly ranges from 27% to 44% among patients with EC within one year of diagnosis (45). The incidence of postoperative depression in this study was 28.2%, which is in line with previously reported rates. Postoperative depression is known to significantly impair quality of life after MIE (46, 47), but its impact on recurrence remains limited. Luo et al. reported that comorbid anxiety and depression independently predicted nutritional impairment and were correlated with significantly inferior survival outcomes compared to nondistressed counterparts in patients with EC during the peri-radiotherapy period (48). The interplay among primary tumor progression, side effects of neoadjuvant therapies and surgery, and depression-induced appetite disturbance may synergistically contribute to malnutrition and poor prognosis through distinct biological pathways (49, 50). From a basic research perspective, depression and EC share commonalities in terms of systemic inflammation and immune dysregulation, suggesting a potentially shared pathogenesis. First, inflammatory cytokines play a prominent role in both conditions. Depression has been linked to elevated levels of cytokines, including TGF-β1, IL-1β and IL-6 (51, 52). These proinflammatory cytokines are also enriched in the TME, contributing to immune suppression and correlating with poor outcomes in patients with ESCC (53, 54). Second, immune cell dysregulation is a hallmark of both depression and EC (55). Elevated serum myeloid-derived suppressor cell (MDSC) expression has been demonstrated in patients with EC, reinforcing the role of these cells in disease progression (56). They not only promote tumor immune escape by decreasing the neutralizing function of T cells (57) but also may be implicated in chronic low-grade systemic inflammation and immune dysfunction in depression (58). Third, immune-inflammatory proteins such as CRP and immune-checkpoint proteins are also involved in depression and EC. Depression and EC were found to be correlated with CRP levels exceeding 10 mg/L (46) and with elevated serum expression, respectively (59). Another classic immune-inflammatory protein is programmed cell death protein 1 (PD-1), which is expressed on the membranes of T-cells and inhibits immune responses by binding to programmed cell death ligand 1 (PD-L1) on cancer cells (53, 60). Patients with EC with increased PD-L1 levels may face a poor prognosis (61, 62). The PD-1/PD-L1 pathway is also dysregulated in patients with depression, leading to reduced immune surveillance and the persistence of inflammatory responses (55). Notably, the immune-inflammatory response affects tumor progression through elevated cytokines, immune−cell dysregulation, and immune−checkpoint activation, which together function as an interconnected cascade. A recent review highlighted that MDSCs, as key immune-suppressive cells, contribute to immune tolerance in the TME by secreting cytokines (such as IL-6 and TGF-β) and upregulating PD-L1 expression, which together inhibit the T-cell-mediated antitumor response and worsen patient prognosis. Simultaneously, tumor-secreted proinflammatory cytokines stimulate myeloid progenitor cells in the bone marrow, leading to their differentiation into MDSCs and recruitment to the tumor site, thereby forming a malignant feedback loop (63). Although the immune-inflammatory response is closely linked to depression, the precise mechanisms involved remain unclear. Future prospective studies should combine mental assessments with corresponding real-time serological data to elucidate the specific mechanism between depression and inflammation and pave the way for a novel scientific issue: whether improvements in mental health could improve prognosis through immune-inflammatory pathways in patients with EC.

Given the relationship between depression and cancer progression, this study also analyzed the risk factors for postoperative depression. LASSO regression identified CPSP as the sole significant factor. Emerging evidence suggests that CPSP is commonly accompanied by depression (64). On the other hand, prolonged depression may enhance central sensitization via long-term potentiation (LTP), potentially exacerbating acute pain after surgery and shifting pain from acute to chronic (65). Given this bidirectional relationship, integrating psychosocial interventions and postoperative pain management may be crucial in optimizing clinical outcomes in patients with EC. Notably, the relationship between postoperative pain and oncological outcomes has increasingly become a focus of research. The National Comprehensive Cancer Network (NCCN) guidelines report that chronic pain is strongly associated with worsened quality of life and prognosis in patients with tumors (66). Furthermore, acute perioperative pain can exacerbate surgical stress responses by increasing sympathetic nervous system activation and neuroendocrine activity, thereby suppressing natural killer (NK) cell cytotoxicity (67). As pivotal antitumor immune effectors, reduced NK cell activity may facilitate the evasion of compromised immunosurveillance by circulating tumor cells, consequently increasing the risk of recurrence and metastasis. While this study did not identify significant associations between postoperative pain and RFS, this does not preclude potential effects within specific patient subgroups. Future longitudinal studies should prioritize clarifying the mechanistic links between CPSP, depression and tumor progression.

The reliability of the prognostic model for RFS was also verified in this study. The prognostic model in patients with EC demonstrated good discriminatory ability, with AUC values of 0.826 and 0.773 for 6-month and 12-month prediction in the training set and 0.817 and 0.789 in the validation set, respectively. Calibration and DCA analyses revealed high accuracy and clinical decision-making value, with the model offering substantial net clinical benefit. This study provides a tool for prognostic evaluation in patients with EC, highlighting the importance of combining psychological interventions with regular follow-up care for oncology treatment in clinical practice.

There are several limitations that we must mention. First, it is a retrospective analysis with a certain degree of selection bias. The data come from a single hospital, which may limit their generalizability. A multicenter, large-sample prospective study for the external validation of our model will be conducted in the future. Second, immune-inflammatory factors and some specific biomarkers at the follow-up time points were unable to be assessed due to the retrospective nature of our study. Larger-scale prospective research is needed to explore the dynamic changes in immune-inflammatory factors and their interactions with psychological health. Finally, although our study fills a gap in research on depression and postoperative survival in patients with EC, the mechanisms linking depression with the prognosis of EC still require further exploration through basic research.

## 5 Conclusion

This study reveals a close relationship between depression, the preoperative SII and the prognosis of patients with EC. Establishing a reliable prognostic model can assist clinicians in identifying high‐risk groups for EC recurrence. These findings provide novel insights for future research to elucidate the specific interaction mechanism of psychiatric comorbidities and prognosis in patients with EC, with the goal of developing targeted therapies.

## Data Availability

The datasets analyzed contain information that could be used to identify the individuals included, therefore are not uploaded as **Supplementary Material**. The datasets can be obtained from the corresponding authors upon reasonable request. Requests to access these datasets should be directed to TY, blizzardyt@163.com.
